# Shuffling effector genes through mini-chromosomes

**DOI:** 10.1371/journal.pgen.1008345

**Published:** 2019-09-12

**Authors:** Li-Jun Ma, Jin-Rong Xu

**Affiliations:** 1 Department of Biochemistry and Molecular Biology, University of Massachusetts Amherst, Amherst, Massachusetts, United States of America; 2 Department of Pathology, Purdue University, West Lafayette, Indiana, United States of America; University of Georgia, UNITED STATES

The rice blast fungus, *Magnaporthe oryzae Oryzae* pathotype (MoO), was listed as the number one fungal plant pathogen [1]. The emerging devastating wheat blast disease that poses a serious threat to global wheat production was caused by *M*. *oryzae Triticum* pathotype (MoT), a different pathotype within the same species complex. Wheat blast was first reported in Brazil in 1985 and rapidly spread to other South American countries, causing significant yield losses. The disease captured public attention in 2016 when it appeared in Bangladesh, resulting in thousands of wheat fields being burned to prevent further spread of the disease [2]. In this issue of *PLOS Genetics*, Peng and colleagues [3] reported the generation and in-depth analysis of a high-quality reference genome of the MoT strain B71, a recent Bolivian isolate highly similar to the MoT strain that has spread to South Asia. Combining the computational skills of the Liu lab and extensive expertise of the Valent lab on *M*. *oryzae* pathogens, the authors identified mini-chromosomes specific to the more aggressive MoT isolates and analyzed their possible roles in adaption to wheat infection.

**Fig 1:**
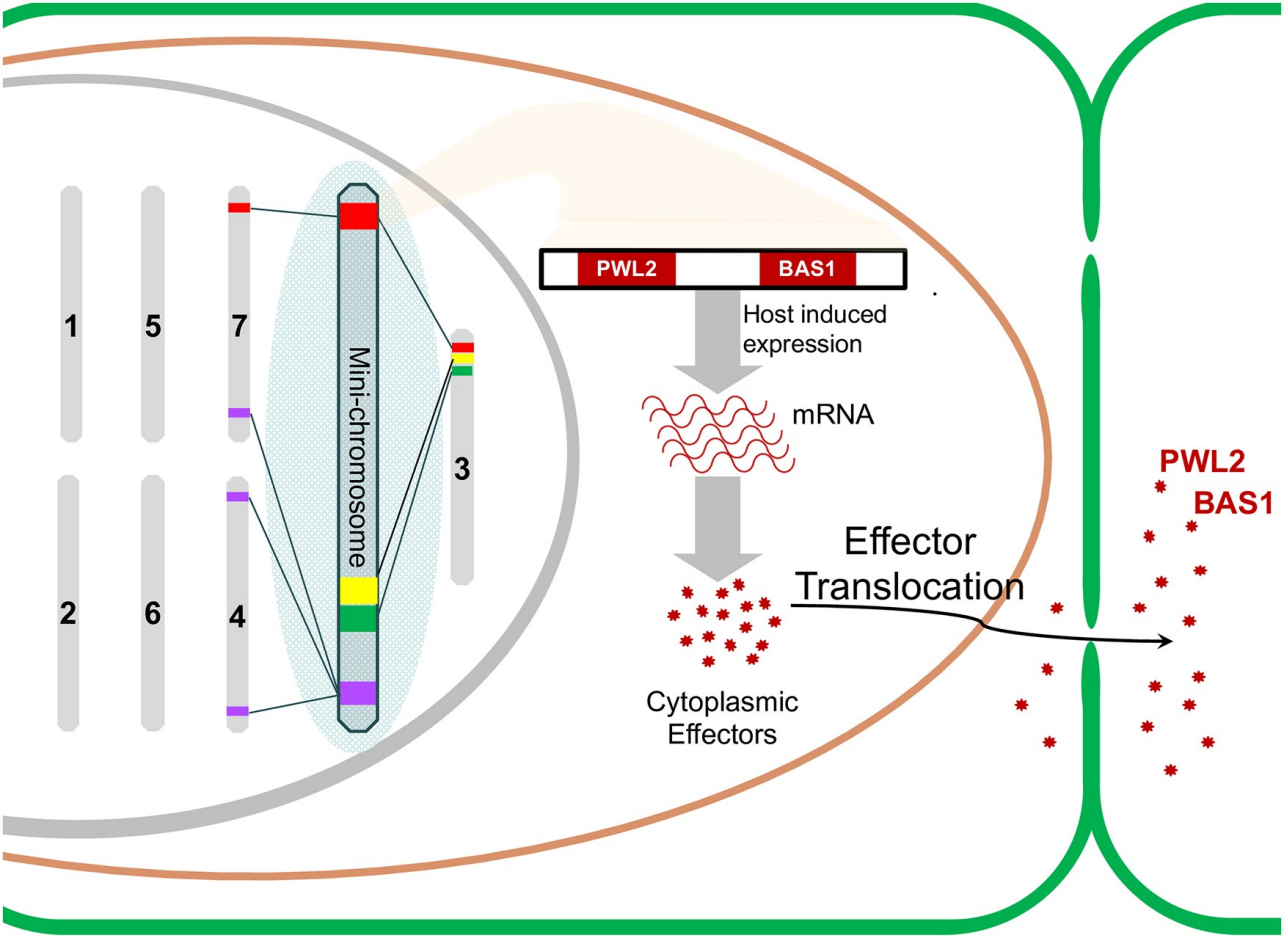
Cartoon illustration of the mini-chromosome, highlighted in light blue background, in the reference genome of *Magnaporthe oryzae Triticum* pathotype (MoT) [3]. While seven core chromosomes, demonstrated as gray bars, are conserved in the rice blast fungus *M*. *oryzae Oryzae* pathotype (MoO), this mini-chromosome is absent. It contains large duplications from ends of core chromosomes and is enriched for effector genes and other genes involved in host invasion. Also illustrated here are two cytoplasmic effectors, *PWL2* and *BAS1*. They are physically linked in the mini-chromosomes, and their expression are induced specifically during wheat infection. As documented in MoO, cytoplasmic effectors effectors can translocate into plant cells via the biotrophic interfacial complex (BIC) and spread to neighboring cells ahead of invasive hyphae [6].

This reference genome was generated using a combination of PacBio, Illumina, and long insert end-pair (LIEP) sequencing. The near complete, high-quality assembly MoT B71 include seven conserved core chromosomes and one mini-chromosome absent in the MoO reference genome [4] (Fig 1). The presence of this mini-chromosome in the B71 genome was confirmed by contour-clamped homogeneous electric field (CHEF) electrophoresis and sequencing analysis. The approximately 2-Mb MoT mini-chromosome lacks house-keeping genes, and 52.8% of its sequences are transposons, a significant enrichment similar to what has been reported in lineage-specific chromosomes in the *Fusarium oxysporum* species complex [5]. Interestingly, transposons enriched in the mini-chromosome are also observed frequently at the ends of core chromosomes. However, unlike most of their counterparts on core chromosomes, transposable elements enriched on the mini-chromosome were not subject to inactivation by extensive repeat-induced point (RIP) mutations.

Large segmental duplications were observed between this B71 mini-chromosome and several ends of core chromosomes (Fig 1). The link between these two compartments of the genome is also reflected by their shared enrichment of effector genes and other genes involved in host invasion. Functionally characterized in the rice blast fungus, both *PWL2* and *BAS1* are cytoplasmic effectors capable of translocating into plant cells via the biotrophic interfacial complex (BIC) and spreading to neighboring cells ahead of invasive hyphae [6]. Interestingly, though these two effectors are located on separate chromosomes in MoO, *PWL2* and *BAS1* are physically linked in the mini-chromosome of MoT, indicating the shuffling of effector genes. The expression of both mini-chromosome–located effectors is specifically induced during wheat infection (Fig 1).

A comparative genomic analysis of six other field isolates of MoT revealed the highly variable nature of mini-chromosomes. While an earlier-emergent and less virulent strain lacks the mini-chromosome, an aggressive strain P3 recently isolated from Paraguay contains two mini-chromosomes. *PWL2* and *BAS1* reside on one of the mini-chromosomes together with the avirulence (*AVR*) gene *AVR-Pib*, and the other mini-chromosome contains only the *AVR-Pik* gene, further indicating dynamic shuffling of effector genes in MoT. *PWL2* has also been reported to be a member of an *AVR* gene family that can be recognized by host plant resistance (*R*) genes in a gene-for-gene manner [7]. The blast fungus is notorious for rapidly overcoming *R* genes deployed in crops, typically by deleting the corresponding *AVR* effector gene. The enrichment and shuffling of effector genes on mini-chromosomes might have provided a reservoir of retained effector genes at the pathogen population level and promoted documented effector gene mobility through movement to new locations in core chromosome ends [8]. This would further increase effector functional diversification, enabling rapid pathogen adaptation.

Among fungal pathogens, effector genes that modulate host immunity are often organized in distinct genomic compartments that are enriched for transposons, enabling genomic flexibility and adaptive evolution [9]. Initially described as conditionally dispensable (CD) or supernumerary chromosomes in the plant pathogen *Fusarium solani* f. sp. *pisi* (synonym *Nectria haematococca*) [10] and then documented at the genomic level in *F*. *oxysporum* [5], mobile mini-chromosomes offer distinct structural and functional compartmentalization within a genome. In addition to reporting the first well-assembled mini-chromosome in *M*. *oryzae* strains, Peng and colleagues [3] demonstrated the connection between the transposon-rich mini-chromosomes and telomeric regions of core chromosomes in MoT. Frequent genetic exchanges between these two parts of the genome can serve as a mechanism for rapid gain or loss of effector and infection-related genes, which, in turn, could accelerate pathogen evolution [3]. This is a significant discovery, although acquisition of homologous genes from ends of core chromosomes only accounts for a fraction of the mini-chromosome sequences.

Overall, the identification of mini-chromosomes and shuffling of effector genes between core and mini-chromosomes are important milestones for improving our understanding of MoT, a rapidly evolving and spreading pathogen. Follow-up functional studies of genes unique to MoT may lead to the identification of virulence factors associated with wheat infection. Because of the dynamic nature of mini-chromosomes and the mobility of effector genes present on these chromosomes, monitoring the genome evolution, particularly the mini-chromosomes, in the MoT strains at the global level is important to better understand the adaptation of this important pathogen and to develop more effective disease management strategies.
